# A Study on the Effects of Bottom Electrode Designs on Aluminum Nitride Contour-Mode Resonators

**DOI:** 10.3390/mi10110758

**Published:** 2019-11-07

**Authors:** Soon In Jung, Chaehyun Ryu, Gianluca Piazza, Hoe Joon Kim

**Affiliations:** 1 Department of Robotics Engineering, Daegu Gyeongbuk Institute of Science & Technology (DGIST), Daegu 42988, Korea; jsi20039@dgist.ac.kr (S.I.J.); chaehyun@dgist.ac.kr (C.R.); 2 Department of Electrical and Computer Engineering, Carnegie Mellon University, Pittsburgh, PA 15213, USA; piazza@ece.cmu.edu

**Keywords:** MEMS, aluminum nitride, resonator, damping, quality factor, electromechanical coupling

## Abstract

This study presents the effects of bottom electrode designs on the operation of laterally vibrating aluminum nitride (AlN) contour-mode resonators (CMRs). A total of 160 CMRs were analyzed with varying bottom electrode areas at two resonant frequencies (*f*_0_) of about 230 MHz and 1.1 GHz. Specifically, we analyzed the impact of bottom electrode coverage rates on the resonator quality factor (*Q*) and electromechanical coupling (*k*^2^), which are important parameters for Radio Frequency (RF) and sensing applications. From our experiments, *Q* exhibited different trends to electrode coverage rates depending on the device resonant frequencies, while *k*^2^ increased with the coverage rate regardless of *f*_0_. Along with experimental measurements, our finite element analysis (FEA) revealed that the bottom electrode coverage rate determines the active (or vibrating) region of the resonator and, thus, directly impacts *Q*. Additionally, to alleviate thermoelastic damping (TED) and focus on mechanical damping effects, we analyzed the device performance at 10 K. Our findings indicated that a careful design of bottom electrodes could further improve both *Q* and *k*^2^ of AlN CMRs, which ultimately determines the power budget and noise level of the resonator in integrated oscillators and sensor systems.

## 1. Introduction

Piezoelectric microelectromechanical systems (MEMS) resonators have shown great promise towards fully integrated and high-efficiency RF and wireless communication systems owing to their small footprint and low power budget [1,2,3,4]. With the emergence of Internet of Things (IoT), the need for ultra-low-power communication and sensor systems is growing, and recently developed piezoelectric MEMS resonator technologies have been demonstrated to be fundamental building blocks in near-zero power sensors for various application areas [5,6,7]. Moreover, many piezoelectric resonators are fully compatible with conventional microfabrication processes, enabling monolithic integration for single-chip electronics [8,9,10]. In contrast to electrostatic resonators [11,12], piezoelectric resonators exhibit better electromechanical coupling and lower motional resistance, which are favorable for 50 Ω RF communication systems.

Among existing piezoelectric MEMS resonators, laterally vibrating aluminum nitride (AlN) contour-mode resonators (CMRs) have drawn much attention as multiple frequency resonators can be fabricated on a single chip [4,13,14]. In more detail, lithographically defined electrodes can realize resonant frequencies (*f*_0_) from MHz to several GHz range, which is a great advantage compared to thin-film bulk acoustic resonators (FBAR) or shear-mode piezoelectric resonators, where resonance is determined by the piezoelectric film thickness [15,16,17]. In addition, AlN CMRs exhibit low motional resistance of around 50 Ω along with a small footprint, which enable ultra-low-power AlN CMR-based complementary metal oxide semiconductor (CMOS) oscillators [18,19]. Although AlN CMRs could be building blocks for next generation RF systems, the quality factor (*Q*) still needs to be improved while maintaining a high electromechanical coupling (*k*^2^) for successful commercialization. The aforementioned properties of the resonator ultimately set the power budget and phase noise performance of the device [20,21,22], and the figure of merit (FOM) of AlN CMRs is defined as the product of the two. 

Previous studies have revealed that damping directly affects both *Q* and *k*^2^ of piezoelectric MEMs resonators [8,23]. In the case of AlN CMRs, both mechanical and thermal effects induce significant damping on the device and these must be accounted for to ensure stable resonator operation. Mechanical damping (or anchor losses) [24] is more dominant in lower-frequency CMRs, while thermoelastic damping (TED) prevails at higher resonant frequencies around or above 1 GHz [25]. Various designs of electrodes, resonators, and anchors, which connect the resonator to the supporting substrate, have been extensively studied in an effort to mitigate such damping effects and, ultimately, improve device performance. In detail, segmented electrodes [26] and apodization techniques [27] have been applied to improve the FOM and suppress spurious modes. Studies on anchors and the bus region, where the top and bottom electrodes do not overlap near anchors, have also been reported to be important factors on mechanical damping, affecting both *Q* and *k*^2^ [28]. Since more than 50% of TED arises from the metal electrodes, the type of electrode material and its thickness also affects the electromechanical properties of AlN CMRs [25,29]. A search for the optimal resonator design and metal electrode continues in an effort to build high performance AlN CMRs with outstanding electromechanical properties. 

All the aforementioned studies have identified the impact of various design parameters on device performance and improved the current state of AlN CMRs. However, there is a lack of published work on the effects of bottom electrode designs on the electromechanical properties of CMRs. In general, a bottom electrode accounts for more than 50% of the metal in a resonator [25,30,31]. In addition, the size of the bottom electrode ultimately determines the active (or vibrating) region of the resonator. Since the design of the bottom electrode can affect both mechanical damping and TED, it is important to understand its impact on device performance. 

To experimentally verify the impact of the bottom electrodes, we fabricated and measured the admittance response of a total of 32 different AlN CMRs with varying anchor designs, bottom electrode coverage rates, and resonant frequencies. In addition, we performed finite element analysis (FEA) to further investigate the impact of electrode designs on mechanical damping in the resonator. To minimize TED, we also measured the resonators at 10K and compared the results to calculations. This work outlines the device design and fabrication, experimental analysis, numerical analysis, and in-depth discussion of the major findings.

## 2. Materials and Methods

### 2.1. Resonator Design and Fabrication

**Figure 1:**
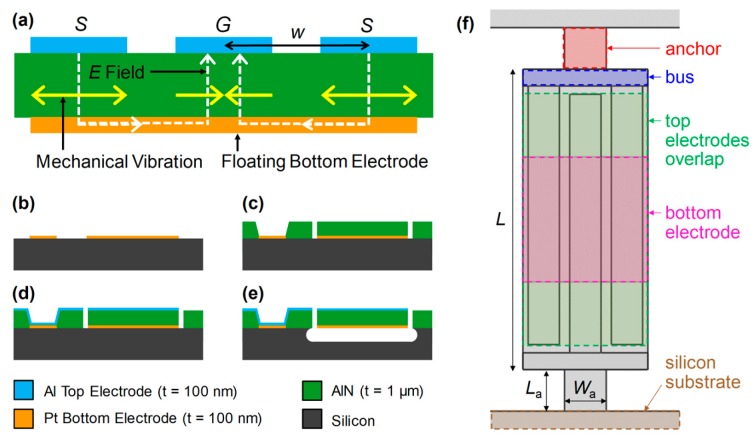
(**a**) Side view of an aluminum nitride (AlN) contour-mode resonator (CMR), which is formed of top electrodes, an AlN layer, and a bottom electrode. (**b**–**e**) The fabrication process consists of a bottom electrode patterning, AlN deposition, top electrode deposition, and the final device release via XeF_2_ etching of a silicon layer. (**f**) A schematic of 220 MHz CMR consisting of the anchor, bus region, top and bottom electrodes, and silicon substrate. The bottom electrode coverage rate is defined as the bottom electrode area (pink) divided by the area where the top electrodes overlap (green).

The designed AlN CMRs consisted of 100 nm thick Al top electrodes, a 1 μm thick AlN piezoelectric layer, and a 100 nm thick Pt bottom electrode, as shown in Figure 1a. The interdigitated top electrodes work as signal and ground pads while the floating bottom electrode guide the electric field vertically. Such an electric field induces lateral vibrations of the resonator. The resonant frequency of the device can be expressed as

*f*_0_ = 1/2*w* × sqrt (*E*_eq_/*ρ*_eq_) (1)

where *w* is the pitch of the top electrodes, *E*_eq_ is the equivalent Young’s modulus, and *ρ*_eq_ is the equivalent mass density of the resonator. Since previous studies have revealed that the type of dominating damping mechanism depends on *f*_0_, we designed a set of CMRs with two different resonant frequencies at 230 MHz and 1 GHz. As anchor losses dominate at lower *f*_0_, we designed the 230 MHz CMRs to have various anchor dimensions. By contrast, the 1.1 GHz CMRs were designed to have full anchors as the impact of anchor losses is rather small compared to TED. In addition, we fabricated CMRs with varying bottom electrode coverage rates ranging from 33% to 120%. Here, 100% was the area where the signal and ground top electrodes overlapped. The bottom electrode coverage rate is defined as the bottom electrode area divided by the area where the top electrodes overlapped, as shown in Figure 1f. For example, a 120% coverage rate means that the bottom electrode covered the entire resonator body including the bus region. For 230 MHz CMRs, the width of the top electrodes were 15 μm with a pitch of 20 μm. For 1.1 GHz CMRs, the top electrodes were 2 μm wide with a pitch of 4 μm. Table 1 summarizes the set of fabricated CMRs with varying design parameters, where *L*_a_, *W*_a_, and *L* are anchor length, width, and resonator length, respectively. In total, we fabricated CMRs with 32 different designs.

**Figure 2:**
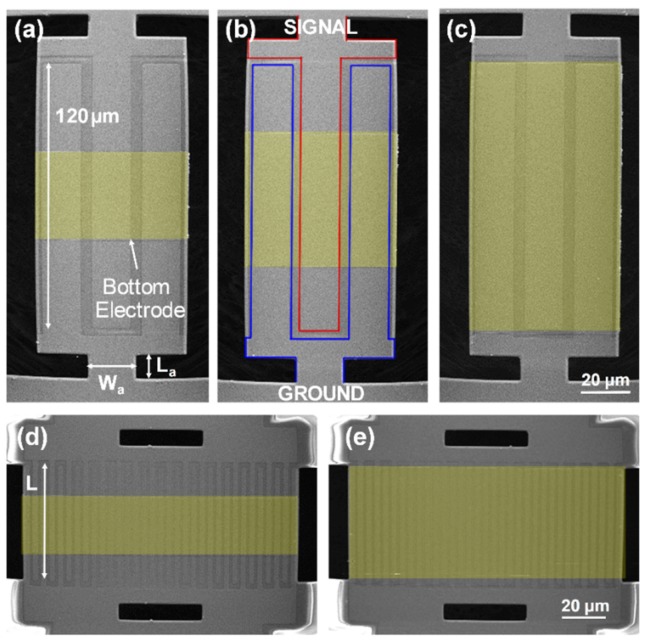
Scanning electron microscope (SEM) micrographs of the released AlN CMRs. (**a**–**c**) 230 MHz CMRs with bottom electrode coverage rates of 33%, 50%, and 100% (highlighted in yellow). (**d**–**e**) 1.1 CMRs with 50% and 100% bottom electrode coverage rates.

Figure 1b shows the overall fabrication process of AlN CMRs. For device fabrication, a 4 inch high resistivity (over 2 × 10^3^ Ω·cm) Si wafer was used to prevent any current leakage from the resonator to the Si substrate. First, a 100 nm thick Pt layer was patterned via liftoff to form the bottom electrodes. Pt was chosen as the bottom electrode material as it allows the growth of c-axis oriented AlN films and can withstand a rather high AlN sputtering temperature of about 400 °C [4]. Then, a 1 μm thick AlN layer was sputter deposited and patterned by photolithography and a reactive ion etching (RIE) process. To form the top electrodes, a 100 nm thick Al layer was patterned and the device was finally released by XeF_2_ etching of the Si substrate. Throughout the fabrication process, we closely monitored and maintained the intrinsic stress of deposited films to be less than ± 100 MPa to prevent any bending after release. The device yield was over 90% and more than 1000 resonators could be fabricated on a single 4 inch wafer. Here, we defined the yield as the number of devices that gave a measurable *Q* and *k*^2^ using a vector network analyzer (VNA) out of the total number of released devices. Figure 2 shows the scanning electron microscope (SEM) images of the released devices. 

### 2.2. Experiment and Finite Element Analysis

The admittance response of AlN CMRs was measured using an RF probing setup and a vector network analyzer. From the measured data, the electromechanical properties, such as *k^2^* and *Q*, were extracted using a modified Butterworth–Van Dyke (mBVD) model. In total, we measured 160 devices corresponding to five devices per each resonator design as presented in Table 1. For room temperature analysis, all the devices were measured at about 293 K. We closely monitored and kept a consistent temperature to remove any temperature effects from the experiment. For low-temperature measurements at about 10 K, a cryogenic probe station was used along with the same data extraction method explained above. By comparing the results from the measurements at 293 and 10 K, we can understand how much TED is affecting device performance.

To understand the impact of bottom electrode designs on the mechanical damping of CMRs, we performed an FEA study using COMSOL Multiphysics software. For the purpose of performing quantitative analysis, we implemented a perfectly matched layer (PML) technique that can predict the mechanical damping and, thus, *Q* of CMRs with reasonable accuracy [23]. However, COMSOL FEA could not be used to predict TED and it was necessary to compare the measurements to predictions when TED is mitigated. Hence, we compared the FEA results to experimental data taken at 10 K, where the effect of any thermoelastic damping were expected to be extremely small in addition to the measurements taken at the ambient temperature of 293 K.

## 3. Results and Discussions

**Figure 3:**
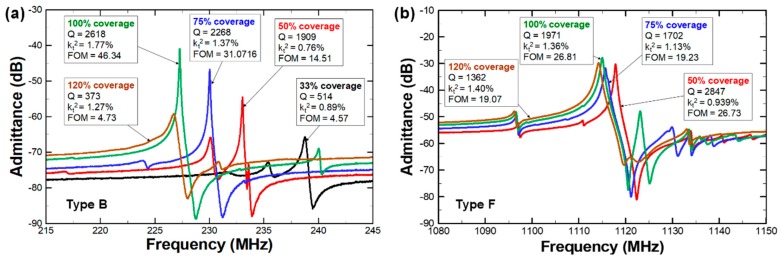
Admittance response of (**a**) 230 MHz and (**b**) 1.1 GHz CMRs. The changes in bottom electrode designs induce significant shift in electromechanical properties of AlN CMRs.

Figure 3 shows the admittance plots of 230 MHz and 1.1 GHz CMRs with varying bottom electrode coverages. Although the resonator size and top electrode configuration remained the same, the change in bottom electrode dimensions induced a significant shift in the admittance response of the resonators. For both frequency devices, *f*_0_ decreased with increasing coverage rates, while *E*_eq_ and *ρ*_eq_ were dependent on the bottom electrode areas. Compared to 230 MHz CMRs, the relative shift in *f*_0_ was smaller for 1.1 GHz devices. The regions with overlapped metal electrodes were substantially larger in terms of acoustic wavelength at 1.1 GHz with respect to the relative coverage at 230 MHz. Thus, changes in the metal overlapping area will have a greater impact on the relative frequency shift of lower-frequency devices than the 1.1 GHz CMRs. For 230 MHz CMRs, the max amplitude of admittance, which directly converts into motional resistance (*R*_m_), also depended on the bottom electrode designs. For 230 MHz CMRs, the shift in *R*_m_ increased by as much as 1040%, while the maximum shift in *R*_m_ was much smaller at 180% for the 1.1 GHz resonators. From the admittance plot analysis, it was noticeable that the impact of bottom electrode designs increased for lower-frequency CMRs.

**Figure 4:**
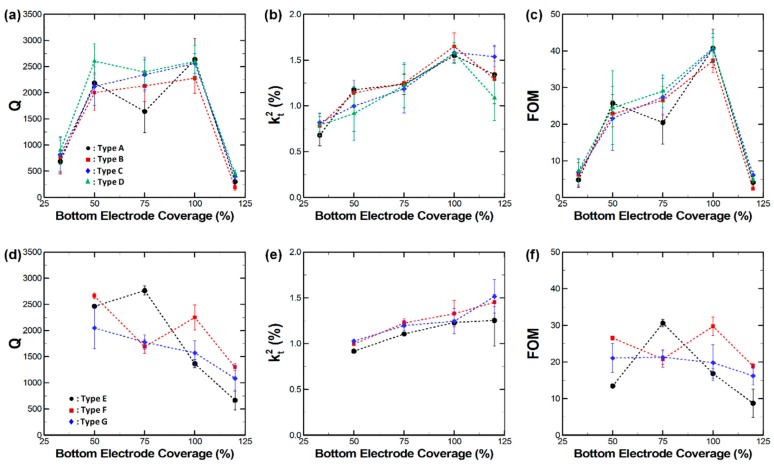
Measured electromechanical properties of (**a**–**c**) 230 MHz and (**d**–**f**) 1.1 GHz CMS with varying bottom electrode coverages. It is clear that both the quality factor (*Q*) and electromechanical coupling (*k*^2^) of CMRs depend on the bottom electrode designs, ultimately affecting the device figure of merit (FOM).

Figure 4 shows the measurement results for *Q*, *k*^2^, and FOM of 230 MHz and 1.1 GHz CMRs as a function of bottom electrode coverage rate. Each data point represents the measurements from five devices of each design. The standard deviation for each measurement is also indicated on the plot. For 230 MHz CMRs, the measured *Q* fluctuated over the studied bottom electrode coverage. Such a trend in *Q* fluctuation was consistent regardless of the differences in anchor designs. By contrast, *Q* decreased with an increasing bottom electrode coverage rate in the case of 1.1 GHz CMRs, which were fabricated with full anchor designs. *k*^2^ values increased with the bottom electrode coverages for both frequency CMRs. This indicates that the electromechanical transduction was more favorable for the larger active region of CMRs. The resulting FOM of CMRs are shown in Figure 4c,f. The FOM peaks were similar to *Q* for 230 MHz devices and remained relatively constant for 1.1 GHz CMRs. 

**Figure 5:**
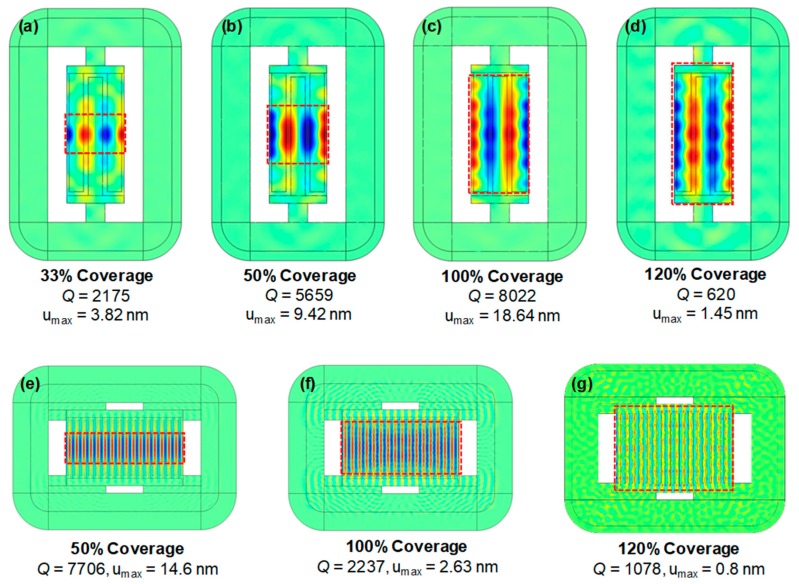
Contour images of vibration displacement fields of (**a**–**d**) Type D 230 MHz CMRs and (**e**–**g**) Type E 1.1 GHz CMRs with varying bottom electrode coverage rates.

The experiment results suggest that the bottom electrode coverage rates impact the device *Q*, or the damping inside the resonator. Specifically, the shift in *Q* was as large as 970% and 392% for 230 MHz and 1.1 GHz CMRs, respectively. To understand the impact of damping on CMRs, we ran FEA using the piezoelectric module and PML methods of the COMSOL Multiphysics software. The PML method accurately predicted the amount of mechanical damping along with the vibration characteristics of the resonators. Figure 5 shows the displacement field along the vibration direction of the resonator at *f*_0_. The expanded region is in red and the compressed is colored blue, which allows a visualization of CMR’s physical motion. The contour images showed that the vibration region of the resonator was strictly limited to the region that was covered with both the top and bottom electrodes. For example, Figure 5a shows that the harmonic vibration of the resonator body was confined in the middle of the bottom electrode covered area. In other words, the significant portion of the region that was covered with the bottom electrode did not vibrate at *f*_0_ for 230 MHz CMRs (Type D) with a 33% bottom coverage rate. However, as the electrode coverage rates increased, the vibrating region fully covered the bottom electrode areas, resulting in improved *Q*. For the 120% bottom coverage, the bus region also had a floating bottom electrode. Thus, the bus region itself worked as a single-finger CMR with a different resonant frequency compared to the region where the top and bottom electrodes overlapped. Such behavior was likely to hinder harmonic oscillation of the resonator and induce large damping, as shown in Figure 5d.

Compared to the 230 MHz devices, the 1.1 GHz CMRs exhibited a trend where *Q* decreased with increasing bottom coverages. Figure 5e shows that the resonator vibration at *f*_0_ was clearly limited to the region covered with a bottom electrode for a 50% coverage. However, an acoustic wave from the resonator body started to propagate towards the supporting Si substrate for higher coverage rates. When the bottom coverage was at maximum (120%), the dissipated wave from the resonator almost reached the PML boundary. Such wave propagations indicate that a significant amount of mechanical energy was being dissipated towards the supporting structures. Although the anchor loss was known to be smaller than other damping mechanisms for high-frequency CMRs [25], our FEA results indicate that the anchor design can still impact damping and such anchor loss should be also be accounted for when considering high-frequency CMRs.

**Figure 6:**
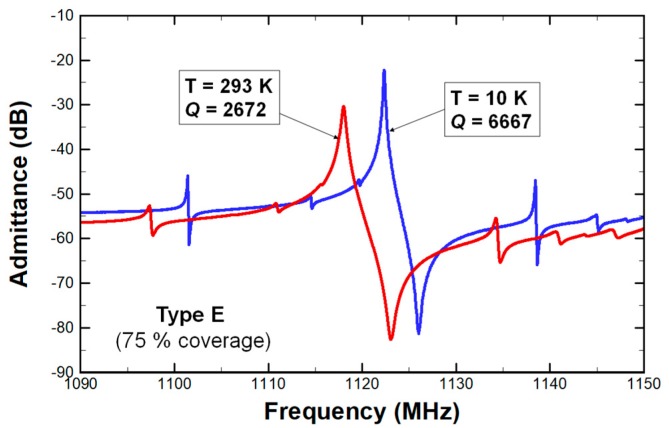
Admittance responses of 1.1 GHz (Type E with 75% bottom electrode coverage) at 293 and 10 K. Both *Q* and resonant frequencies (*f*_0_) depend on operating temperatures.

To validate the FEA results, we measured the 230 MHz and 1.1 GHz CMRs at a cryogenic temperature of 10 K, where TED was largely mitigated. Hence, the *Q* of CMR was mostly limited by anchor losses or other mechanical damping effects. Figure 6 shows the admittance responses of an identical resonator at 10 and 293 K. The device *Q* improved by about 250% by eliminating TED. A previous study involving TED analysis [25] reported a comparable decrease in unloaded *Q* at lower operating temperatures. Thus, we can conclude that such large drop in *Q* is largely due to the mitigated TED at 10 K. In addition, the device *f*_0_ shifted by about 5.7 MHz because the material properties of the resonator were dependent on operating temperatures. 

**Figure 7:**
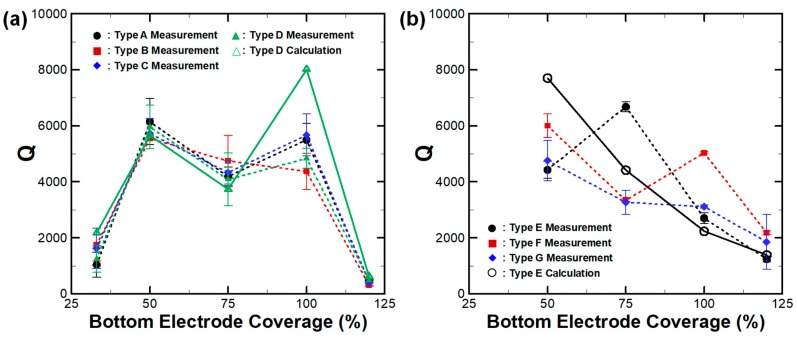
Measured (at 10 K) and predicted *Q* of (**a**) 230 MHz and (**b**) 1.1 GHz CMRs with varying bottom electrode coverages at 10 K. For all measured resonators, *Q* drastically improved by eliminating the thermoelastic damping (TED) effect. The prediction follows the general trends of the measurements.

In addition to the measurements at 290 K, all 160 CMRs measured at 10 K and *Q* had significantly improved for both 230 MHz and 1.1 GHz devices, as shown in Figure 7. Quantitatively, *Q* increased by about 239% and 208% for 230 MHz and 1.1 GHz CMRs, respectively. Such a large enhancement of *Q* indicates that the impact of TED is significant for CMRs regardless of operating frequencies, as previously reported [25]. Although TED was largely mitigated at 10 K, *Q* followed the trends observed at 293 K and we can assume that such fluctuations in the electromechanical properties of CMRs were largely affected by mechanical damping effects. The predicted *Q* of Type D and Type E CMRs were compared to the measured data. Their values followed a similar trend with reasonable accuracy, further validating our FEA approach. A rather large discrepancy between the measurement and the prediction was present for Type D devices with 100% bottom electrode coverage. In such cases, we assumed the actual device experienced additional sources of damping which were neither anchor losses nor TED.

From the measurement and FEA of CMRs with varying bottom electrode coverage rates, it is obvious that the electromechanical properties, such as *Q* and *k*^2^, were impacted by the bottom electrode configurations. Both parameters were important as they affected both the power budget and noise level of CMR-integrated oscillators. In addition, *Q* ultimately sets the limit of detection (LOD) of AlN CMR-based sensors. Our results suggest that a shift in the bottom electrode designs induce changes in the amount of mechanical damping or anchor loss. By contrast, the impact of bottom electrode coverage rates on TED was rather small, as the ratio of metals to piezoelectric material in the active regions of the resonator remained constant. Since most CMRs or other types of piezoelectric MEMS resonators operate at temperature ranges where TED is present, it was important to optimize and account for the impact of the bottom electrode designs to alleviate mechanical damping in an effort to enhance the overall device performance.

The findings from this work can contribute to the enhancement of the current state of AlN CMR-based sensors. Such sensors utilize a functional layer (such as a magnetic material or chemically absorbing interface) on top of the resonator to detect a targeted event with outstanding sensitivity and LOD [32,33,34,35,36,37]. In addition, AlN CMRs with plasmonic nanostructures have been developed for spectrally selective infrared sensing [38]. For the aforementioned sensing applications, a certain portion of the resonator surface is covered or patterned with a functional layer, which cannot effectively work as a metal electrode. Since the sensor in [38] has a floating electrode with partial coverage, similar to the cases covered in this work. As we investigated the impact of varying electrode coverage rates, our results can be extended to determine the optimal ratio between the metal electrodes and a functional layer, or to predict the electromechanical properties of CMR-based sensors. More broadly, the results of this study can be applied to CMRs with interdigitated bottom electrodes and a floating top electrode.

## 4. Conclusions

We reported the impact of bottom electrode designs on electromechanical properties, such as *Q*, *k*^2^, and FOM of 230 MHz and 1.1 GHz AlN CMRs. Both measurements and FEA calculation results showed that the change in bottom electrode coverage rates impact CMRs. Such an effect is largely due to the change in the amount of mechanical damping, which was more dominant in lower-frequency CMRs, and led to large fluctuations in *Q*. For 1.1 GHz CMRs, *Q* decreased with increasing bottom electrode areas as the damping towards supporting substrate increased. To validate our claims, we measured CMRs at 10 K, confirming that the experimental results match the theoretical predictions. Compared to mechanical damping or anchor loss, the effect of TED remained constant regardless of changes in bottom electrode designs. This is because TED depends on the ratio of metal-to-piezoelectric material coverage only in the active region of the resonator. We believe our findings can contribute to the enhancement of the current state of AlN CMRs and possibly other types of piezoelectric MEMS resonators by improving their electromechanical properties. These finding will directly impact applications in which CMRs are used as the main components, such as in oscillator and sensor systems.

## Figures and Tables

**Table 1:** Parameters of fabricated 230 MHz and 1.1 GHz CMRs with various anchor designs and bottom electrode coverage rates.

230 MHz CMRs				1.1 GHz CMRs		
Type	Anchor Length (L_a_)	Width (W_a)_	Bottom Electrode Coverage	Type	Resonator Length (L)	Bottom Electrode Coverage
A B C D	10 μm 10 μm 20 μm 20 μm	10 μm 20 μm 10 μm 20 μm	33%, 50%, 75%, 100%, 120%	G E F	60 μm 80 μm 100 μm	50%, 75%, 100%, 120%
